# Whole exome sequencing-based homologous recombination deficiency test for epithelial ovarian cancer

**DOI:** 10.1186/s13048-024-01565-3

**Published:** 2025-01-30

**Authors:** Ying-Cheng Chiang, Hsien-Neng Huang, Kuan-Ting Kuo, Wuh-Liang Hwu, Po-Han Lin

**Affiliations:** 1 Department of Obstetrics and Gynecology, College of Medicine, National Taiwan University, Taipei City, Taiwan; 2 Department of Obstetrics and Gynecology, National Taiwan University Hospital, Taipei City, Taiwan; 3 Department of Obstetrics and Gynecology, National Taiwan University Hospital Hsin-Chu Branch, Hsinchu, Taiwan; 4 Department of Pathology, National Taiwan University Hospital Hsin-Chu Branch, Hsinchu, Taiwan; 5 Department of Pathology, College of Medicine, National Taiwan University, Taipei City, Taiwan; 6 Department of Pediatrics, National Taiwan University Hospital, Taipei City, Taiwan; 7 Department of Medical Genetics, National Taiwan University Hospital, 19F, No. 8, Chung-Shan South Road, Taipei City, Taiwan; 8 Graduate Institute of Medical Genomics and Proteomics, College of Medicine, National Taiwan University, Taipei City, Taiwan

**Keywords:** Epithelial ovarian cancer, Homologous recombination deficiency test, Whole-exome sequencing, scarHRD

## Abstract

**Background:**

The homologous recombination deficiency (HRD) test is an important tool for identifying patients with epithelial ovarian cancer (EOC) benefit from the treatment with poly(adenosine diphosphate-ribose) polymerase inhibitor (PARPi). Using whole exome sequencing (WES)-based platform can provide information of gene mutations and HRD score; however, the clinical value of WES-based HRD test was less validated in EOC.

**Methods:**

We enrolled 40 patients with EOC in the training cohort and 23 in the validation cohort. The WES-based HRD score was calculated using the scarHRD software. We first evaluated the concordance of the HRD status defined by the Myriad MyChoice CDx and then assessed the value of HRD on clinical prognosis in patients with EOC.

**Results:**

The HRD score defined by the WES-based test was positively correlated with that of the Myriad MyChoice^®^ CDx test (*r* = 0.82, *p* < 0.01) in the training cohort. In compared to HRD status of Myriad test, the sensitivity, specificity, positive predictive value, and negative predictive value of the WES-based HRD test were 93.5% (29/31), 77.8% (7/9), 93.5% (29/31), and 77.8% (7/9), respectively. Patients with positive HRD status defined by WES-based scarHRD test and Myriad MyChoice^®^ CDx test were both highly associated with platinum sensitive response (both Fisher’s exact test, *p* = 0.002) as well as the superior progression-free survival (both log-rank *p* = 0.002). The multi-variate Cox regression model incorporated with optimal debulking surgery showed that the recurrence risk was decreased in the patients with positive HRD status, either defined by Myriad MyChoice^®^ CDx test (Hazard ratio (HR) 0.33, 95% confidence interval (CI) 0.14–0.79, *p* = 0.013) or WES-based test Myriad MyChoice^®^ CDx test (HR 0.34, 95% CI 0.14–0.80, *p* = 0.014). Nine patients had mutations in the genes involved in HR DNA repair, and all of them were positive for HRD. In the validation group, 23 patients were defined as positive HRD by WES-based testing. Six positive HRD patients and 5 negative HRD patients received maintenance PARPi. The median responsive interval of PARPi was 17 months in positive HRD patients and 3 months in negative HRD patients.

**Conclusion:**

The WES-based test is a potential option for determining the HRD status in EOC patients, and desires for further validation in large-scale cohorts.

**Supplementary Information:**

The online version contains supplementary material available at 10.1186/s13048-024-01565-3.

## Introduction

Epithelial ovarian cancer (EOC) is a major cause of cancer-related death in women [1, 2]. Owing to the lack of specific symptoms and screening tools for identifying early-stage disease, most EOC patients are diagnosed at an advanced stage, where the disease would have spread beyond the pelvis, with a 5-year survival of less than 50% [3]. Most advanced-stage EOC patients relapse after a good response to primary treatments, including debulking surgery and adjuvant platinum-based chemotherapy, and the response to salvage chemotherapy is generally unsatisfactory, leading to poor prognosis [3, 4].

Precision medicine is an evolving area in EOC that depends on the distinct genetic or molecular features of cancer, which are the targets of therapy. Maintenance PARPi therapy is a good example of an EOC [5, 6]. Accumulation of DNA damage caused by replication errors, oxidative stress, ultraviolet light, radiation, or cytotoxic agents can lead to genomic instability. DNA damage response (DDR) pathways repair single-strand breaks (SSBs) or double-strand breaks (DSBs) in damaged DNA and DDR dysfunction is associated with carcinogenesis [7]. Homologous recombination repair (HRR) is an error-proof DNA repair pathway involving several genes, such as *BRCA1/2*, which restores the original sequence at the DSB sites [7]. Homologous recombination deficiency (HRD) is a condition in which when HRR is impaired, DSBs are repaired by error-prone pathways, such as non-homologous end joining (NHEJ), single-strand annealing, or microhomology-mediated end joining, which causes errors in the sequence of the repaired DNA [7]. Poly (adenosine diphosphate-ribose) polymerase (PARP) participates in SSBs repair by binding to DNA strand breaks. PARP inhibitor (PARPi) therapy is based on “synthetic lethality,” in which DSBs induced by PARPi are repaired by error-prone pathways, leading to genomic instability and subsequent apoptosis in HRD cancer cells [8].

The dilemma is that the most promising target drugs benefit only in a subpopulation, and it is important to select the right patients for targeted therapy. A cost-effective analysis suggested that PARPi should be reserved for EOC patients with positive HRD status [9, 10]. To stratify EOC patients for PARPi is currently based on the HRD status, and Myriad MyChoice^®^ CDx test was one commercial test suggested by US Food and Drug Administration (FDA) and European Medicines Agency (EMA) [5, 6]. HRD is a highly sensitive biomarker to PARPi or platinum-based chemotherapy [11, 12]. However, the currently available FDA-approved HRD tests are not economical and feasible for real-world applications; establishing a method that most laboratories can perform is more consistent with clinical needs.

A recent concept has shifted from single-nucleotide polymorphism (SNP) sequencing to whole-genome sequencing (WGS) or whole-exome sequencing (WES) [13]. Compared to SNP sequencing, WES provides more useful information about actionable genetic variants, microsatellite instability, and even tumor mutational burden for immune checkpoint blockade therapy. Some studies have demonstrated a very good correlation in HRD status between Myriad MyChoice^®^ CDx testing and the WGS/WES method for breast cancer [13, 14]. However, the clinical significance of WES-based HRD analysis for EOC has not yet been validated.

In this study, we used a training cohort to develop a WES-based HRD test for EOC patients and evaluated its concordance with the results of the Myriad MyChoice^®^ CDx test in the HRD status. We also assessed the prognostic and predictive value of HRD, which was defined using a WES-based test in EOC patients. We then confirmed the performance of the HRD test in the validation cohort to demonstrate its ability to guide PARPi therapy.

## Methods

### Patients and specimens

**Fig. 1:**
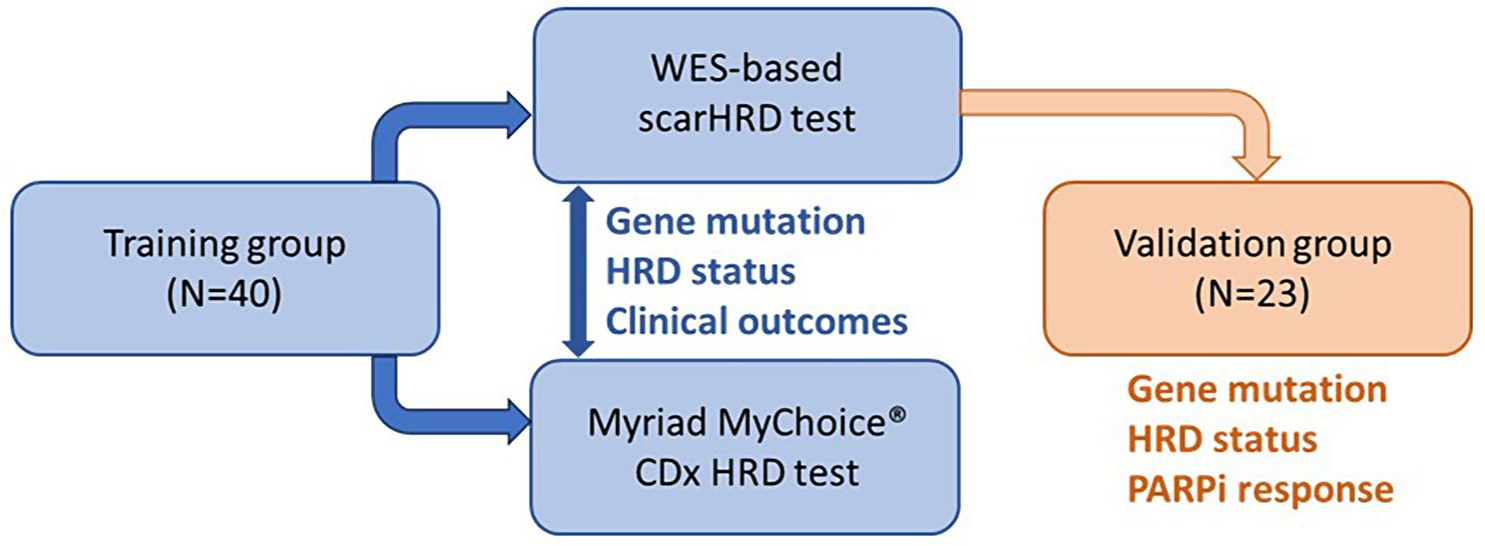
The flowchart of the study. In training group, the gene mutation and HRD status of 40 EOC patients was examined by our WES-based scarHRD test and Myriad MyChoice^®^ CDx test. The correlation of clinical outcomes and HRD status were analyzed. In validation group, the gene mutation and HRD status of 23 EOC patients was defined by our WES-based scarHRD test. The correlation of clinical outcomes, especially the PARPi response, and HRD status were analyzed

The study protocol was approved by the National Taiwan University Hospital Research Ethics Committee (201807116RINA), and the study was performed in accordance with the guidelines and regulations. As shown in Figs. 1 and 40 patients were included in the training and validation groups, respectively. Specimens were retrieved from formalin-fixed paraffin-embedded (FFPE) tissues obtained from the primary debulking surgery. After pathologists’ review, FFPE specimens with a tumor purity of more than 20% were sliced at a thickness of 5–10 μm and sent for experiments.

Clinical data were obtained from patients’ medical records, including age, cancer stage, tumor grade, residual tumor size after debulking surgery, pathological reports, adjuvant treatments, and outcomes. All the patients underwent primary debulking surgery and adjuvant platinum-based chemotherapy. For debulking surgery, R0 resection was defined as no gross residual tumor following surgery, R1 resection as a maximal residual tumor size of < 1 cm following surgery, and R2 resection as a maximal residual tumor size *≥* 1 cm. Stage was defined based on the International Federation of Gynecology and Obstetrics (FIGO) criteria, and tumor grade was defined based on the International Union Against Cancer criteria [15]. Cancer recurrence was defined as biopsy-proven disease, abnormalities reported in imaging studies (including computed tomography or magnetic resonance imaging), or continuously elevated levels of cancer antigen 125 (CA-125; more than twice the upper normal limit) for at least two consecutive tests with a monthly interval. Patients were designated as “platinum-sensitive” when the tumor recurs beyond 6 months after completing primary treatment, and “platinum-resistant” when the tumor recurred within 6 months after completing primary treatment. Progression-free survival (PFS) was defined as the interval from the date of completion of the primary treatment to the date of confirmed recurrence, progression, or last follow-up. Overall survival (OS) was defined as the interval from the date of diagnosis to the date of EOC or the last follow-up.

### DNA extraction and library preparation

Genomic DNA was isolated from FFPE specimens using a Quick-DNA FFPE extraction kit (Zymo Research, CA, USA), according to the manufacturer’s instructions. A total of 100-ng ng DNA per sample was used for library preparation. DNA fragmentation and library construction were performed using the KAPA HyperPlus Kit for next-generation sequencing (NGS) DNA Library Preparation. An exome library was generated using Roche KAPA HyperExome Probes (Roche, Basel, Switzerland).

### Sequencing and bioinformatics

The samples were sequenced using Illumina NovaSeq with paired-end reads of 300 nucleotides, and the analysis algorithm was in accordance with our previous protocol [16]. Raw sequencing data were aligned with the reference human genome (December 2013, GRCh38) using Burrows-Wheeler Aligner software (version 0.5.9) [16]. The SAM tools (version 0.1.18) were used for data conversion, sorting, and indexing [16]. We used the Genome Analysis Toolkit (GATK; version 4) Mutect2 for variant calling, including nonsynonymous variants, small insertion/deletions (indels), and variants of splicing boundaries [16]. After variant calling, ANNOVAR was used for annotation of the genetic variants [16, 17]. ClinVar, dbSNP (version 150), Exome Sequencing Project 6500 (ESP6500), and 1000 Genomes variant datasets (ExAC and gnomAD) were used to filter common variants in the sequencing results. Pathogenic/likely pathogenic variants, which were defined by guidelines for the interpretation of sequencing variants, were considered deleterious and used for further analysis [18]; variants of uncertain significance were not enrolled. In the WES-based test, we focused on pathogenic variants of genes involved in DDR pathways, including *BRCA* genes (Supplementary Table S1) [16, 19].

### WES-based HRD test

**Fig. 2:**
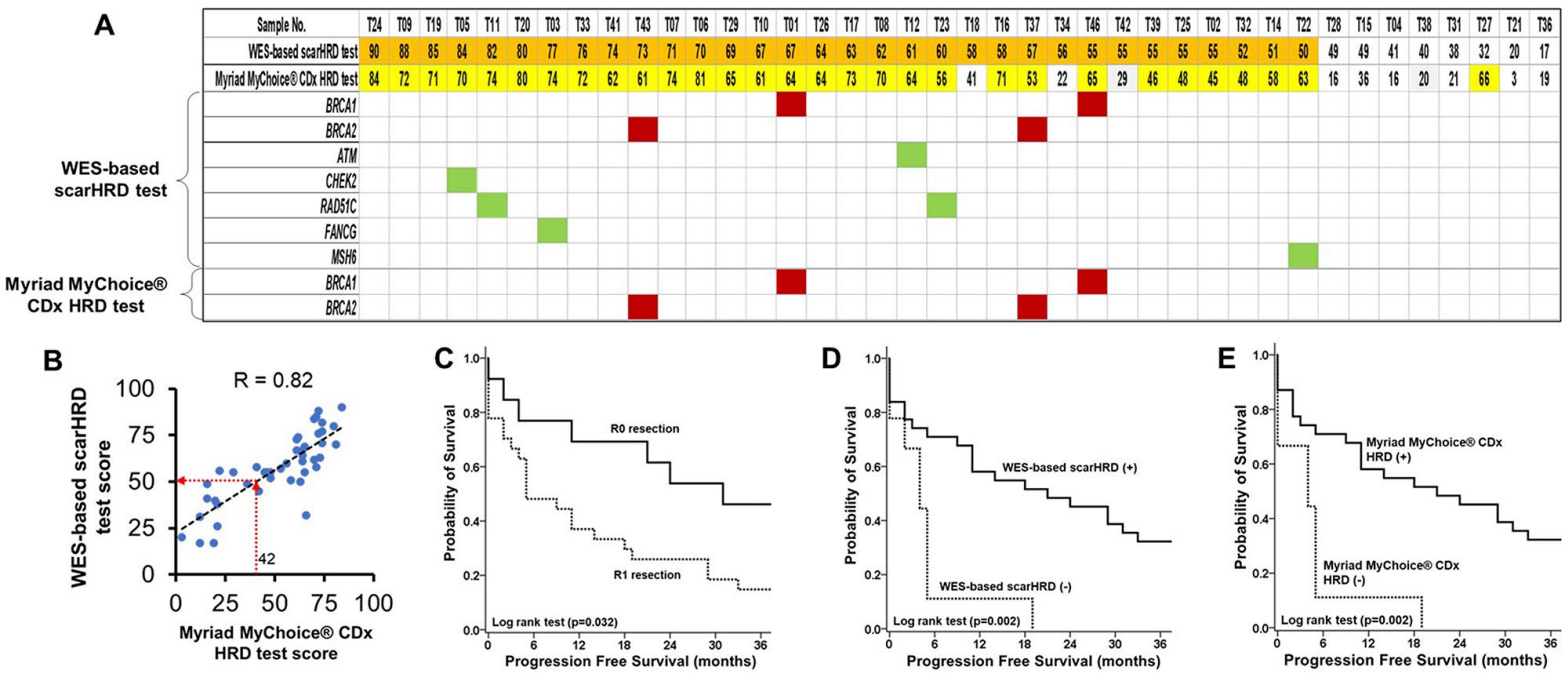
The HRD scores, gene mutations and Kaplan-Meier analysis of progression free survival in training group of EOC. (**A**) The HRD status. *Note*: The numbers indicate the HRD score from the two tests. The orange color indicates a positive HRD status obtained using our WES-based scarHRD test. The yellow color indicates a positive HRD status obtained using the Myriad MyChoice^®^ CDx test. The red color indicates *BRCA1/2* gene mutations. The green color indicates other DDR gene mutations. (**B**) Correlation between the WES-based scarHRD score and the Myriad MyChoice^®^ CDx test score. *Note*: WES-based scarHRD test score is highly correlated with the MyChoice^®^ CDx test score. A MyChoice^®^ CDx test score of 42 is equal to the WES-based scarHRD score of 50 obtained using the linear regression model. (**C**) PFS of EOC patients stratified by surgical resection status. *Note*: EOC patients who underwent R0 resection had better PFS than those who underwent R1 resection (*p* = 0.032, log-rank test). (**D**) PFS of EOC patients stratified by our WES-based scarHRD test. *Note*: EOC patients with a positive HRD status had better PFS than those with a negative HRD status (*p* = 0.002, log-rank test). (**E**) PFS of EOC patients stratified by Myriad MyChoice^®^ CDx test. *Note*: EOC patients with a positive HRD status had better PFS than those with a negative HRD status (*p* = 0.002, log-rank test)

We used scarHRD software to measure the HRD score, which is a combination of loss of heterozygosity (LOH) [11], the number of chromosomal regions with allelic imbalance extending to the telomere (TAI) [20], and large-scale state transition (LST) [21]. The LOH score was defined as the number of LOH regions greater than 15 Mb in length. TAI refers to the unequal contribution of parental allele sequences extending to the end of the chromosome. LST is defined as a chromosomal break between adjacent regions, each of which is at least 10 Mb, and the distance between them is not larger than 3 Mb. The scarHRD is an R package program downloaded from the website (https://github.com/sztup/scarHRD) [13]. The positive HRD status of the specimen was defined under the following two conditions. First, deleterious variants of BRCA1 or BRCA2 were detected. Second, the HRD score of the specimen calculated using scarHRD software was *≥* 50, which reflected the score of 42 of the Myriad MyChoice^®^ CDx test by linear regression analysis (Fig. 2B).

### Myriad MyChoice® CDx test

The Myriad MyChoice^®^ CDx test is an NGS-based diagnostic test that is conducted using DNA isolated from FFPE specimens. This test performed (1) qualitative detection of single-nucleotide variants (SNVs), insertions and deletions (indels), and large genomic rearrangement variants in protein-coding regions and intron/exon boundaries of *BRCA1*/*2* genes and (2) determination of the genomic instability score (GIS), which is an algorithmic measurement of LOH, TAI, and LST. Positive HRD status was defined as pathogenic or likely deleterious mutations in *BRCA1/2* or GIS *≥* 42.

### Statistical analysis

The chi-square test and Fisher’s exact test were used to calculate significant differences in the variables between the groups. The sensitivity and specificity of the WES-based scarHRD test were assessed using the Myriad MyChoice CDx test as a reference. The correlation between the HRD status and clinical outcomes was analyzed. PFS and OS were estimated using Kaplan-Meier analysis and log-rank tests. The HRD status on the risk of recurrence and death was evaluated using univariate and multivariate Cox proportional hazards regression models with corresponding 95% confidence intervals (CI). All p values were two-sided, and less than 0.05 were considered statistically significant.

## Results

### Clinicopathologic characteristics of the training cohort

There were 40 patients with EOC in the training group, and their clinical characteristics are shown in Table 1. The median age was 56.5 years old, and the median pretreatment CA-125 value was 889 U/ml. All patients had advanced-stage (FIGO stages III and IV) high-grade serous EOC. All patients underwent primary debulking surgery, with R0 resection in 13 and R1 resection in 27. All patients received adjuvant platinum-based and paclitaxel chemotherapy, and 23 (57.5%) patients were platinum-sensitive. The median follow-up duration was 60 months. The Tumor recurred in 31 (77.5%) patients, and 21 (52.5%) patients died due to EOC.

**Table 1:** Characteristics of epithelial ovarian cancer patients in the training cohort

Exercise group	
Number of patients	40
Age (years old)	56.5(39–75)
Pretreatment CA-125 (U/ml)	889(153–7561)
Histology: Serous carcinoma	40(100%)
FIGO stage: Advanced	40(100%)
Tumor grade: high	40(100%)
Debulking surgery	
R0	13(32.5%)
R1	27(67.5%)
Platinum response	
Sensitive	23(57.5%)
Resistant	17(42.5%)
Recurrence	
Yes	31(77.5%)
No	9(22.5%)
Death	
Yes	21(52.5%)
No	19(47.5%)

Note: Data of age and CA-125 are presented as median (minimum-maximum), whereas those of other parameters are presented as number (percentage). CA-125, cancer antigen 125; FIGO, International Federation of Gynecology and Obstetrics

### Establish the of WES-based HRD

The HRD scores and deleterious gene mutations detected in these two tests are shown in Fig. 2A. In the WES-based scarHRD test, the HRD scores of 40 patients ranged from 17 to 90. In the Myriad MyChoice CDx test, the HRD scores of 40 patients ranged from 3 to 84. A linear regression model was applied to analyze the correlation between the WES-based scarHRD score and the Myriad MyChoice CDx HRD score (Fig. 2B). The WES-based scarHRD score was strongly correlated with the Myriad MyChoice CDx × HRD score (correlation coefficient (r): 0.82, *p* < 0.001). Based on the regression model, we defined positive HRD status as *BRCA* gene mutation or a score of ≥ 50 in our WES-based scarHRD test, which is equal to a score of 42 in the Myriad MyChoice^®^ CDx test.

Overall, 32 patients had a positive HRD status (score *≥* 50) in the WES-based test and 30 patients had a positive HRD status according to the Myriad MyChoice^®^ CDx test (score *≥* 42). For DDR gene mutations, *BRCA1* mutation was noted in two patients, *BRCA2* in two patients, *ATM* in one patient, *CHEK2* in one patient, *RAD51C* in two patients, *FANCG* in one patient, and *MSH6* in one patient (Supplementary Table S2). All patients with DDR mutations had a positive HRD status. In addition, we confirmed that the sequencing outcome of *BRCA* mutations in the cohort found by the Myriad MyChoice^®^ CDx test was recapitulated in our WES-based test.

Compared with the positive HRD status in the Myriad MyChoice^®^ CDx test, the sensitivity, specificity, positive predictive value (PPV), and negative predictive value (NPV) of the WES-based HRD test were 93.5% (29/31), 77.8% (7/9), 93.5% (29/31), and 77.8% (7/9), respectively (Supplementary Table S3).

### Correlation of HRD status to clinical outcomes in the training group

In WES-based test, a higher percentage of patients with a platinum-sensitive response had a positive HRD status than that of patients with a platinum-resistant response (95.7% [22/23] vs. 52.9% [9/17], *p* = 0.002, Fisher’s exact test, Table 2). Similarly, a higher percentage of patients with a platinum-sensitive response had a positive HRD by Myriad MyChoice^®^ CDx test than that of patients with a platinum-resistant response (95.7% [22/23] versus 52.9% [9/17], *p* = 0.002, Fisher’s exact test, Table 2). There was no significant difference in the percentage of EOC patients with positive HRD status as defined by the two tests according to cancer recurrence and cancer-related death.

**Table 2:** Correlation of HRD status and clinical parameters of EOC patients in the training group

HRD positive status		Platinum response		Recurrence		Death	
Total		Sensitive	Resistant	No	Yes	No	Yes
40		23	17	9	31	19	21
WES-based scarHRD test							
Negative	9	1(4.3%)	8(47.1%)	0(0%)	9(29%)	3(15.8%)	6(28.6%)
Positive	31	22(95.7%)	9(52.9%)	9(100%)	22(71%)	16(84.2%)	15(71.4%)
p value*			0.002		0.090		0.457
Myriad MyChoice^®^ CDx HRD test							
Negative	9	1(4.3%)	8(47.1%)	0(0%)	9 (29%)	4(21.1%)	5(23.8%)
Positive	31	22(95.7%)	9(52.9%)	9(100%)	22(71%)	15(78.9%)	16(76.2%)
p value*			0.002		0.090		1.000

Note: HRD, homologous recombination deficiency; WES, whole-exome sequencing

*Fisher’s exact test

**Fig. 3:**
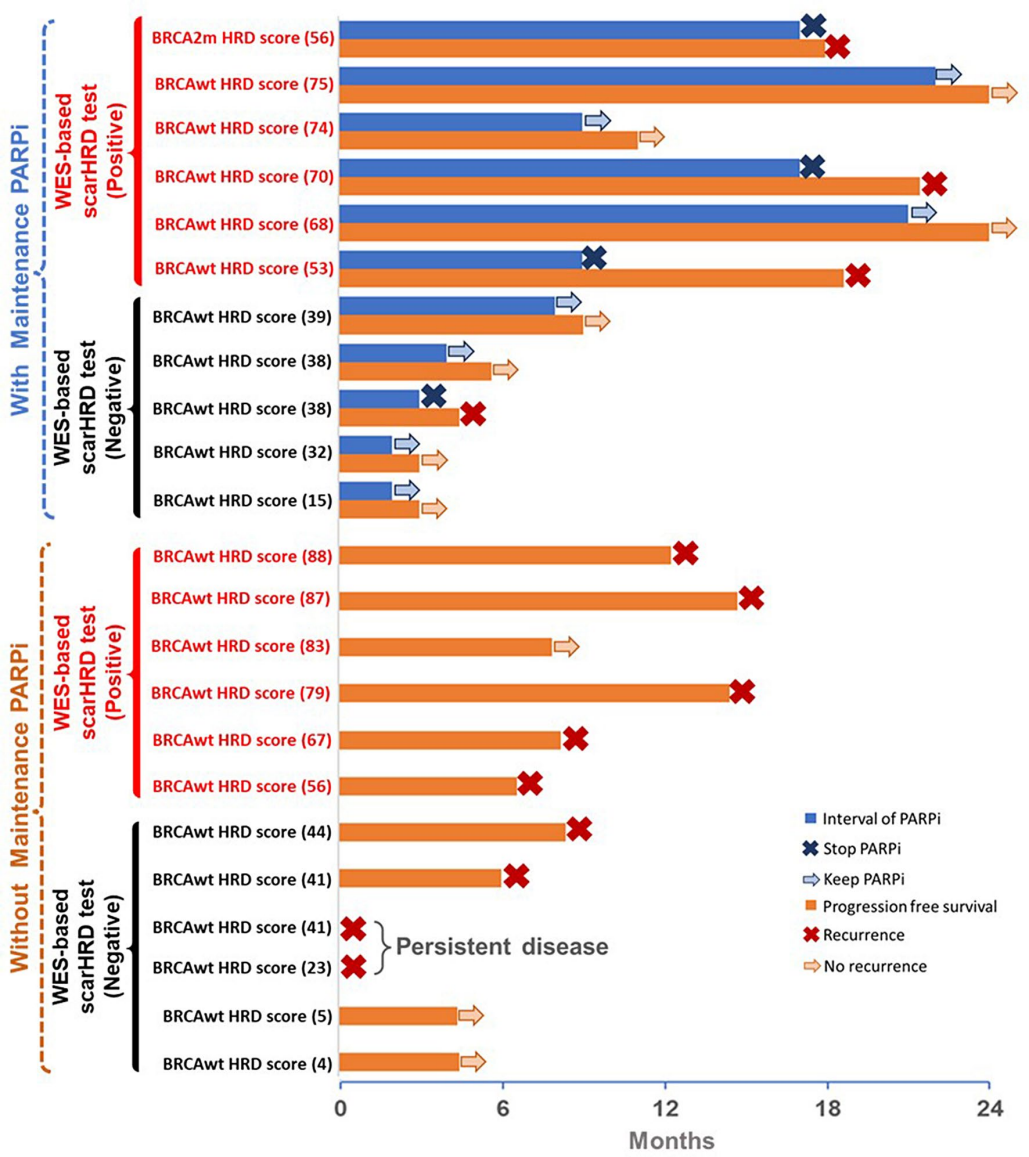
The HRD status and clinical outcomes in validation group of EOC. The median intervals of PARPi in positive HRD EOC patients were longer than negative HRD patients. The median PFS of positive HRD EOC patients were longer than negative HRD patients. *Note*: The blue row indicated the interval of the patient taking PARPi. The orange row indicated progression free survival of the patient. The dark blue cross indicated the patient stopped taking PARPi. The light blue arrow indicated the patient keep PARPi. The red cross indicated cancer recurrence or persistent disease. The light red arrow indicated no evidence of cancer recurrence

Patients with EOC who underwent debulking surgery with R0 resection had a longer PFS (*p* = 0.032, log-rank test; Fig. 2C) and OS (*p* = 0.013, log-rank test; Supplementary Fig. 1A) than those who underwent R1 resection. Patients with a positive HRD status, either by the WES-based test or the Myriad MyChoice^®^ CDx test, had a longer PFS (both *p* = 0.002, log-rank test; Fig. 2D and E). The predictive value of OS for the two HRD tests was unsatisfactory (Supplementary Fig. 1B, C).

Debulking surgery with R0 resection (hazard ratio [HR] 0.42, 95% CI 0.18–0.98, *p* = 0.045) and HRD positive status test (WES based HRD: HR 0.29, 95% CI 0.12–0.68;, Myriad MyChoice CDx test: HR 0.28, 95% CI 0.12–0.66) were associated with disease recurrence in univariate analysis. The multivariate Cox regression model showed that a positive HRD status, which was defined by either the WES-based test or the Myriad MyChoice^®^ CDx test, was an independent factor for disease progression after adjustment for R0/R1 resection. However, a positive HRD status in both tests was associated with a better OS trend, which was not statistically significant. The multivariate Cox regression model for the risk of cancer-related death revealed that only debulking surgery with R0 resection was an independent risk factor in the multivariate analysis (See Table 3).

**Table 3:** Cox regression model for evaluating the risk factors for recurrence and death in EOC patients of training group (*n*=40)

	*n*	Recurrence				Death			
		Univariate		Multivariate		Univariate		Multivariate	
		H.*R*. (95% C.I.)	*p*	H.*R*. (95% C.I.)	*p*	H.*R*. (95% C.I.)	*p*	H.*R*. (95% C.I.)	*p*
Debulking surgery									
R1	27	1 (reference)		1 (reference)		1 (reference)		1 (reference)	
R0	13	0.42(0.18-0.98)	0.045	0.48(0.20-1.16)	0.104	0.24(0.07-0.82)	0.023	0.26(0.07-0.90)	0.033
WES-based scarHRD test									
Negative	9	1 (reference)		1 (reference)		1 (reference)		1 (reference)	
Positive	31	0.29(0.12-0.68)	0.005	0.34(0.14-0.80)	0.014	0.40(0.14-1.12)	0.080	0.49(0.17-1.37)	0.170
Debulking surgery									
R1	27	1 (reference)		1 (reference)		1 (reference)		1 (reference)	
R0	13	0.42(0.18-0.98)	0.045	0.49(0.20-1.18)	0.112	0.24(0.07-0.82)	0.023	0.25(0.07-0.88)	0.031
Myriad MyChoice^®^ CDx HRD test									
Negative	9	1 (reference)		1 (reference)		1 (reference)		1 (reference)	
Positive	31	0.28(0.12-0.66)	0.004	0.33 (0.14-0.79)	0.013	0.40(0.14-1.19)	0.099	0.48(0.16-1.43)	0.190

### Evaluation of WES-based scarHRD test in validation group

There were 23 patients in the validation group, all of whom had advanced-stage high-grade serous EOC. The follow-up period was 24 months. The clinical characteristics are shown in Table 4. Twelve patients were positive for HRD status, as defined by the WES-based test. The median age was 57.5 years old in patients with a positive HRD status and 64 years in those with a negative HRD status. The median pretreatment CA-125 level was 1211 U/ml in patients with positive HRD and 1206 U/ml in those with negative HRD. All patients underwent primary debulking surgery, with R0 resection in three patients, R1 in six patients, and R2 in three patients with positive HRD. Among the negative HRD patients, R0 was observed in six patients, R1 in three patients, and R2 in two patients. All patients received adjuvant platinum-based and paclitaxel chemotherapy, and platinum-sensitive response was noted in 12 (100%) patients with positive HRD patients and three (27.2%) of negative HD patients. As shown in Fig. 3, six positive and five negative HRD patients received maintenance PARPi. The median interval of PARPi was 17 months in patients with positive HRD and 3 months in those with negative HRD results. Eight patients with positive HRD and five with negative HRD had cancer recurrence, and the median PFS was 14.5 months in patients with positive HRD and 4 months in patients with negative HRD.

**Table 4:** Characteristics of epithelial ovarian cancer patients in the validation cohort

Validation group		
WES-based scarHRD test	Positive	Negative
Number of patients	12	11
Age (years old)	57.5(46–77)	64(28–74)
Pretreatment CA-125 (U/ml)	1211 (375- 10000)	1206 (227-17750)
Histology: Serous carcinoma	12(100%)	11(100%)
FIGO stage: Advanced	12(100%)	11(100%)
Tumor grade: high	12(100%)	11(100%)
Debulking surgery		
R0	3(25%)	6(54.5%)
R1	6(50%)	3(27.3%)
R2	3(25%)	2(18.2%)
Platinum response		
Sensitive	12(100%)	3(27.2%)
Resistant	0(0%)	4(36.4%)
N.A.	0(0%)	4(36.4%)*
Patient numbers with PARPi	6(50%)	5(45.5%)
PARPi interval (months)	17(9–22)	3(2–8)
Recurrence		
Yes	8(66.7%)	5(45.5%)
No	4(33.3%)	6(54.5%)
Progression free survival (months)	14.5(8–24)	4(0–9)

Note: Data of age, CA-125, PARPi interval and progression free survival are presented as median (minimum-maximum), whereas those of other parameters are presented as number (percentage). CA-125, cancer antigen 125; FIGO, International Federation of Gynecology and Obstetrics

*The follow-up interval after completing primary treatments in 4 patients was less than 6 months, and therefore it could not be categorized by platinum response

## Discussion

In our proof-concept study, we demonstrated a high degree of consistency in HRD assessment using the WES-based method and the Myriad MyChoice^®^ CDx test. Patients with positive HRD status were associated with platinum sensitivity and better PFS than those without HRD. We then validated the performance of the WES-based HRD test in patients with advanced EOC for maintenance PARPi therapy, which showed satisfactory outcomes, suggesting that the WES-based HRD test is a potential tool for EOC patients.

The WES-based scarHRD test provides an HRD score representing genomic instability to determine the HRD status of patients with EOC. “Genomic scars” represent a record that reflects the repair of DNA damage in response to harmful exposure through multiple pathways in cells [22]. Currently available methods for detecting “genomic scars” use SNP-based microarray or NGS to measure large-scale structural lesions in tumor specimens, including LOH, TAI, and LST [23]. The above three are characteristics of impaired DNA repair activity for DSBs, and the genomic instability score (GIS) combines them to represent the degree of HRD-related genomic instability. Myriad MyChoice^®^ CDx (Myriad Genetics) and Foundation Focus CDx *BRCA* LOH (Foundation Medicine) are Food and Drug Administration-approved diagnostic HRD tests. Positive HRD status in the Myriad MyChoice^®^ CDx test was determined by *BRCA* mutation or GIS ≥ 42 in PAOLA-1 [24] and PRIMA [5], and by *BRCA* mutation or GIS ≥ 33 in VELIA [25]. The cutoff value of GIS was determined retrospectively from exploratory analyses, and these PARPi trials were not prospectively designed to stratify patients by HRD tests. The Foundation HRD test includes *BRCA* mutations and genomic LOH, calculated as the fraction of genomic regions with LOH by sequencing SNPs in tumor specimens. Moreover, 14% genomic LOH was considered a positive HRD status in the ARIEL2 trial [26], whereas 16% genomic LOH was considered the threshold in the ARIEL3 trial [27]. The compatibility between HRD defined by GIS and the percentage of genomic LOH also needs to be determined.

Recently, the concept of HRD testing has shifted from SNP-based methods to WGS or WES methods. For example, HRDetect, a WGS-based assay, could predict *BRCA* deficiency with sensitivity of 98.7% and nearly 100% in 560 breast cancer cases and 73 ovarian cancer cases, respectively, in the validation cohort [28]. However, the ability of HRDetect to predict the PARPi response in EOC has not been confirmed [14, 29]. WGS analyzes the whole genome, whereas WES analyzes all coding regions, which comprise 1-2% of the genome [30, 31]. The original data provided by WGS are quite larger than those provided by WES; therefore, WGS is more time-consuming and expensive than WES [32, 33]. Thus, WES is more affordable in clinical practice if WES-based HRD is accurate. The scarHRD is an open-source R program that can be freely downloaded, and WGS or WES data can be used to calculate GIS. The results of our WES-based scarHRD test correlated well with those of the Myriad HRD test, and the WES-based scarHRD test provided a predictive value for clinical outcomes. These findings suggested that the potential of our WES-based HRD test to help selecting EOC patients for PARPi. The cutoff value for HRD in our WES-based scarHRD test was 50, which was different from that of 42 for the Myriad MyChoice^®^ CDx test. The use of different methods and a baseline reference to measure the HRD score may generate different thresholds to define a positive HRD status [34]. The linear regression model revealed a highly positive correlation between WES-based scarHRD and Myriad HRD scores. Thus, we defined the threshold of our WES-based scarHRD score according to the results of the linear regression analysis when the Myriad HRD score was equal to 42. The positive HRD status defined by our test had acceptable sensitivity, specificity, and PPV/NPV compared to the HRD status determined by the Myriad MyChoice^®^ CDx test. EOC patients with a positive HRD status according to the WES-based test showed a sensitive response to platinum chemotherapy, favorable PARPi maintenance interval, and better PFS. It suggested that the clinical utility of our WES-based scarHRD test for EOC is encouraging.

The WES-based test provides information about DDR gene mutations, including BRCA and non-BRCA DDR genes, such as *ATM*, *BRCA1/2*, *BRIP1*, *MLH1*, *MSH2*, *MSH6*, *PALB2*, *RAD51C*, and *RAD51D*, as recommended by the National Comprehensive Cancer Network guidelines for EOC [35]. The percentage of *BRCA1/2* somatic mutations in serous EOC in this study was 7.9% (5/63), consistent with the findings of previous literature [36, 37]. Approximately 11–18% of high-grade serous EOC patients have a germline *BRCA* mutation and another 6–7% of patients with somatic *BRCA* mutations can be identified from tumor specimens [36, 37]. In addition to *BRCA* mutated EOC patients who receive the greatest benefit from PARPi therapy, patients with non-*BRCA* HR gene mutations such as *ATM*, *BRIP1*, *PALB2*, *RAD51C*, *RAD51D* also derive a survival benefit [37, 38]. Furthermore, these genes are cancer-predisposing genes, and somatic sequencing of pathogenic variants of the above genes may imply a potential germline origin [39]. Genetic counseling and cancer prevention are suggested if genetic testing confirms germline origin. Thus, WES is helpful in providing comprehensive management for patients with EOC.

The present study had limitations. First, the number of cases in our proof-of-concept study was small, and it is necessary to recruit more participants to confirm the value of WES-based scarHRD test in further study. Second, no prospective randomized trial-designed clinical response to PARPi was observed in this cohort. In the training group, we used platinum sensitivity as the clinical surrogate marker to develop our WES-based scarHRD test because platinum sensitivity has been used as an indicator for obtaining GIS [11, 20, 21, 23]. In the validation group, it showed the potential of our WES-based scarHRD test to select EOC patients for PARPi. However, the median follow-up duration was only 24 months, which was insufficient to evaluate long-term benefits. In the future, it is necessary to conduct a prospective long term follow-up study to validate the feasibility of our WES-based scarHRD test to guide PARPi in a larger and more diverse populations of EOC patients. Third, we compared the WES-based scarHRD test only with the Myriad test in the study. We choose Myriad test as reference because both our WES-based test and Myriad test had similar algorithm to calculate HRD score which evaluates LOH, TAI, and LST. In the future study, it is necessary to compare with another FDA-approved Foundation Focus CDx BRCA LOH test to provide a more comprehensive evaluation of the performance of the WES-based HRD test.

In conclusion, the WES-based test provided both information on gene mutations and HRD scores. Based on the proof-of-concept study, the WES-based scarHRD test is a potential option for HRD testing in EOC patients, and further large-scale study is needed to validate the clinical applicability.

## Electronic supplementary material

Below is the link to the electronic supplementary material.

Supplementary Fig. 1. Kaplan-Meier analysis of overall survival in training group of EOC. (A) OS of EOC patients stratified by surgical resection status. *Note*: EOC patients who underwent R0 resection had better OS than those who underwent R1 resection (*p* = 0.013, log-rank test). (B) OS of EOC patients stratified by our WES-based scarHRD test. *Note*: No significant difference was noted in EOC patients with a positive or negative HRD status. (C) OS of EOC patients stratified by Myriad MyChoice^®^ CDx test *Note*: No significant difference was noted in EOC patients with a positive or negative HRD status

## Data Availability

No datasets were generated or analysed during the current study.

## Abbreviations

DDR: DNA damage response
DSBs: Double-strand breaks
EMA: European Medicines Agency
EOC: Epithelial ovarian cancer
FDA: Food and Drug Administration
FFPE: Formalin-fixed, paraffin-embedded
FIGO: International Federation of Gynecology and Obstetrics
GIS: Genomic instability score
HR: Homologous recombination
HRD: Homologous recombination deficiency
HRR: Homologous recombination repair
Indels: Insertions and deletions
LOH: Loss of heterozygosity
LST: Large-scale state transition
NGS: Next-generation sequencing
NHEJ: Non-homologous end joining
NPV: Negative predictive value
OS: Overall survival
PARP: Poly (adenosine diphosphate-ribose) polymerase
PARPi: Poly (adenosine diphosphate-ribose) polymerase inhibitor
PFS: Progression-free survival
PPV: Positive predictive value
SNP: Single-nucleotide polymorphism
SNVs: Single-nucleotide variants
SSBs: Single-strand breaks
TAI: Telomere allelic imbalance
WES: Whole exome sequencing
WGS: Whole-genome sequencing
