# Supplementary material for: Whole exome sequencing-based homologous recombination deficiency test for epithelial ovarian cancer
Source: J Ovarian Res. 2025 Jan 30;18:19. doi: 10.1186/s13048-024-01565-3 (PMC11780812; doi:10.1186/s13048-024-01565-3)
Supplement: Supplementary file 1 — Supplementary Fig. 1. Kaplan-Meier analysis of overall survival in training group of EOC. (A) OS of EOC patients stratified by surgical resection status. Note: EOC patients who underwent R0 resection had better OS than those who underwent R1 resection (p = 0.013, log-rank test). (B) OS of EOC patients stratified by our WES-based scarHRD test. Note: No significant difference was noted in EOC patients with a positive or negative HRD status. (C) OS of EOC patients stratified by Myriad MyChoice® CDx test Note: No significant difference was noted in EOC patients with a positive or negative HRD status [file 13048_2024_1565_MOESM1_ESM.pdf]

Figure S1

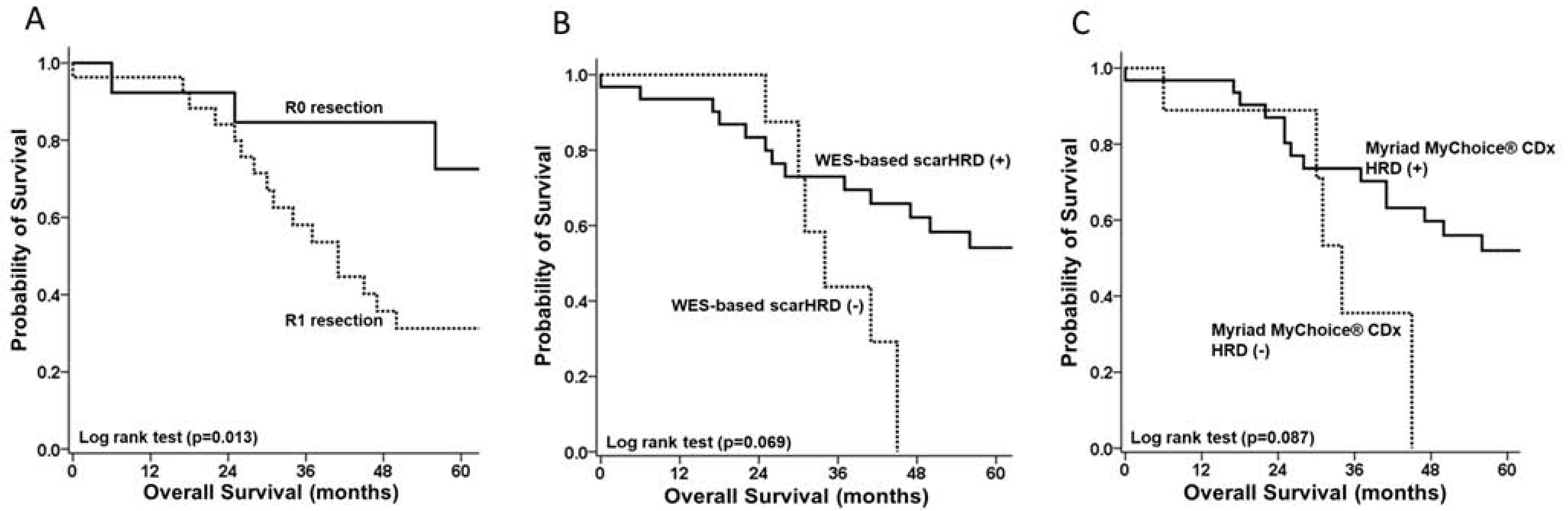
A
B
C
R0 resection
R1 resection
Log rank test (p=0.013)
WES-based scarHRD (+)
WES-based scarHRD (-)
Log rank test (p=0.069)
Myriad MyChoice® CDx HRD (+)
Myriad MyChoice® CDx HRD (-)
Log rank test (p=0.087)
Probability of Survival
Overall Survival (months)

Supplementary Table S1. List of DNA damage response genes

BER	HR	MMR	NER	NHEJ	HR&NHEJ	TLS
MUTYH	*ATM*	*MLH1*	*DDB1/XPE*	*ku70/XRCC6*	*MRE11*	*POLH*
OGG1	*CHEK2*	*MLH3*	*DDB2/XPE*	*ku80/XRCC5*	*NBN*	*POLK*
POLB	*BRCA1*	*MSH2*	*ERCC1*	*XRCC2*	*RAD50*	
	FANCA	*MSH3*	*ERCC2/XPD*	*XRCC3*		
	FANCB	*MSH6*	*ERCC3/XPB*	*XRCC4*		
	FANCC	*PMS1*	*ERCC4*	*NBN*		
	FANCD1/BRCA2	*PMS2*	*ERCC5*			
	FANCD2		*ERCC6/CSB*			
	FANCE		*ERCC8/CSA*			
	FANCF		*XPA*			
	FANCG/XRCC9		*XPC*			
	FANCI		*POLD1*			
	FANCJ/BRIP1		*POLE*			
	FANCL/PHF9					
	FANCM					
	FANCN/PALB2					
	FANCO/RAD51C					
	FANCP/SLX4					
	RAD51					
	RAD51D					

BER: base-excision repair, HR: homologous recombination, MMR: mismatch repair, NER: nucleotide-excision repair, NHEJ: non-homologous end-joining; TLS: translesion synthesis

Supplementary Table S2: The DDR gene mutations of epithelial ovarian cancer patients in exercise group detected by WES-based scarHRD test

Gene	Accession number	Mutation
ATM	NM_000051	exon43:c.6260delA:p.N2088Ifs*8
BRCA1	NM_007300.3	del exon 14
BRCA1	NM_007300	exon10:c.G2635T:p.E879X
BRCA2	NM_000059	exon11:c.2170delA:p.V726Ffs*4
BRCA2	NM_000059	exon11:c.4410_4413del:p.K1472Tfs*6
CHEK2	NM_001349956	exon12:c.1250delC:p.P417Rfs*8
FANCG	NM_004629	exon14:c.G1828T:p.E610X
MSH6	NM_001281492	exon5:r.spl
RAD51C	NM_058216	exon7:c.905-2A>C
RAD51C	NM_058216	exon7:c.905-2A>C

Table S3. The HRD-positive status between the WES-based scarHRD test and Myriad MyChoice® CDx HRD test of EOC patients in the training cohort

Myriad MyChoice® CDx HRD test		WES-based scarHRD test			
		Total	Positive	Negative	
		40	31	9	Sensitivity: 93.5%
	Positive	31	29	2	Specificity: 77.8%
	Negative	9	2	7	PPV: 93.5%
	P value*			<0.001	NPV: 77.8%

Note: HRD, homologous recombination deficiency; NPV, negative predictive value; PPV, positive predictive value; WES, whole-exome sequencing.
*Fisher's exact test.
